# Geographic Translocation of Bats: Known and Potential Problems

**DOI:** 10.3201/eid1309.020104

**Published:** 2003-01

**Authors:** Denny G. Constantine

**Affiliations:** * State of California Department of Health, Richmond, California, USA

**Keywords:** geographic translocation, bats, rabies, lyssaviruses, histoplasmosis, perspective

## Abstract

Natural, accidental, and intentional translocation of bats, both intra- and intercontinentally, has been documented. Some bats have been translocated while incubating infectious diseases, including rabies or related lyssavirus infections; others have escaped confinement en route to or at their destinations, while others have been released deliberately. Known events and potential consequences of bat translocation are reviewed, including a proposed solution to the attendant problems.

Among the many potential consequences resulting from the geographic translocation of life forms is the spread of infectious disease organisms harbored by that life form. This consequence was demonstrated long ago by the early devastation of native American human populations caused by pathogens inadvertently introduced by European explorers. Similarly, wildlife rabies outbreaks occurred recently in the United States after foxes, coyotes, and raccoons were translocated to restock areas where these animals are hunted for sport. Wild populations of introduced species can also become common disease vectors where few or none existed before, such as the current role of Indian mongooses (*Herpestes javanicus*) in rabies transmission on Caribbean islands (*1*), or they can become predators of native species, for example, the wildlife destruction that occurred after ferrets and stoats were introduced into New Zealand (*2*).

Bats have been translocated through natural, accidental, and deliberate means. Pathogens associated with bats, such as *Rabies virus* (RABV) and related lyssaviruses (*3*–*6*), can cause disease after protracted incubation periods, ensuring the extended survival of the host and parasite during periods of translocation. Many bat species enter a hibernationlike state in a cold environment, which further prolongs survival. In this article, I describe some occurrences of bat translocation (published, as well as previously unreported) and the potential consequences of that translocation, as the basis for suggesting preventive measures to alleviate the problems that accompany the relocation of bats across the world.

## Translocation of Bats

### Natural Translocation

Some species of bats hibernate at the approach of cold weather; other species migrate to warm areas instead. Bats that migrate along coastlines take shortcuts over water and are apparently blown far out to sea at times. Many North American migrant bats have been found in Bermuda, 1,046 km east of North Carolina, United States, during fall and spring migrations, evidently having been blown there by wind along with waves of migratory birds (*7*). These translocated bats include Hoary Bats *(Lasiurus cinereus),* Red Bats *(L. borealis),* Seminole Bats *(L. seminolus*), and Silver-haired Bats (*Lasionycteris noctivagans*), all species from which RABV has been isolated (*8*). Hoary Bats are also occasionally found in rabies-free Iceland, also possibly blown there by the wind; one bat was captured in the Orkney Islands, off rabies-free Scotland (*9*). Similarly, Hoary Bats are sometimes found in the Galapagos Islands, 966 km off the west coast of South America (*10*).

### Translocation after Landing on Ships

Exhausted bats flying far at sea both individually and in flocks have been reported to alight on ships and be transported to unintended destinations. Most records from the North Atlantic Ocean involve Red Bats and Silver-haired Bats (*11*). A Southern Yellow Bat (*Lasiurus ega*) landed on a ship over 322 km from the coast of Argentina (*12*). A “fruit-destroying bat” was reported sleeping in the rigging of a ship upon arrival in Hawaii from the Philippines (*13*), and a frugivorous bat (*Vampyressa pusilla*) evidently boarded a vessel passing through the Panama Canal and was later found aboard when the ship was between Australia and Tasmania (*14*).

### Translocation after Using Ships for Shelter

Bats sometimes roost in or on ships in port and may be transported as a consequence. Silver-haired bats were discovered hibernating in hulls of ships, and numbers of them found various refuges on ships and yachts in New York (*15*). Little Brown Bats (*Myotis lucifugus*) roosted aboard a ship that frequently traveled from Canada to Europe, flying ashore after arrival in the Netherlands and England (*16*). The presence of individual Little Brown Bats in rabies-free Iceland (*9*) and Kamchatka, Russia (*17*), has been attributed to travel by ship. RABV, other viruses, and *Histoplasma capsulatum* have been found in this species (*3*,*8*).

On January 21, 1997, a stevedore working in the hold of a ship being unloaded in Long Beach, California, after its arrival from Korea, was bitten on the back of his neck by a bat. A fluorescent rabies antibody test was negative for RABV infection. On February 1, I received the bat for evaluation and determined it to be a Serotine bat *(Eptesicus serotinus),* which is similar to the Big Brown Bat (*E. fuscus*) but with a slightly more massive skull. The Serotine has been reported in North Africa and England and across Europe and Asia to Korea. Hundreds of ill or dead Serotines have been found infected with the RABV-related *European bat lyssavirus 1* (EBLV-1) in Europe, where one or two persons have died of the infection after bat bites (*5*). The rabies conjugate used in the rabies test on the Serotine bat’s brain reportedly reacts with this virus as well.

### Translocation in Shipping Containers

Translocation of bats by ship also occurs when bats are closed inside shipping containers. Free-tailed bats from the tropics are occasionally transported long distances in fruit shipments (*18*). A Pallid Bat (*Antrozous pallidus*) was discovered in Victoria, British Columbia, in a shipment of lettuce from California (*19*), where RABV-infected Pallid Bats have been identified. A Big Brown Bat was found hibernating in a timber container from Canada when it was unloaded in the Netherlands (*16*). An Asiatic Pipistrelle bat *(Pipistrellus javanicus)* was discovered in a container transported by ship from Japan to New Zealand (*20*). Sasaki et al. (*21*) reported the arrival in rabies-free Hawaii of a RABV-infected Big Brown Bat found flying in an automobile container from California. Subsequent study indicated that previously the bat had been transported to California either from Florida in the shipping container or from Michigan in an automobile.

In October 1995, a group of live bats was observed hanging in a dark corner within a large shipping container that had just arrived at a Los Angeles port from Puerto Rico, but the bats escaped as capture was attempted and no further reports of these bats were made. Histoplasmosis, apparently absent in California except for imported human infections, has been diagnosed in some Puerto Rican bats.

### Translocation by Aircraft

Bat translocation by aircraft has been reported several times. A Little Brown Bat was found clinging to a seat in an airplane at the end of a flight in Canada (*22*). An Eastern Pipistrelle bat (*Pipistrellus subflavus)* was recovered from a plane that had just arrived in Texas from Mexico (*23*); RABV-infected bats of this species have been identified in the United States and Canada. The carcass of a Little Brown Bat, presumably from Tacoma, Washington, was found on a runway at an Air Force base on rabies-free Guam (*24*). Stebbings reported the arrival in England of a Silver-haired Bat aboard a U.S. Air Force cargo plane from Delaware (*25*). Observed flying in the plane en route, the bat was captured later while sleeping in a crew member’s bed in the aircraft.

An Asiatic Pipistrelle bat was captured May 25, 1993, aboard an airliner en route from Tokyo to San Francisco. This bat was negative for RABV. The next month a Yuma Myotis bat (*Myotis* *yumanensis)* was discovered flying aboard a U.S. Air Force cargo plane en route from California to Hawaii. This bat was also negative for RABV, although rabies has been diagnosed in the species in California. Evidently the bat was loaded into the aircraft within a shipment of fruit.

In early March 1995, a traveler who had just arrived in Los Angeles by aircraft from South Africa opened his suitcase and observed a bat fly out. The suitcase had been closed three days earlier during darkness in a hut within Kruger National Park. The bat was negative for RABV, and the frozen carcass was sent to me 2 months later with the history of origin in a Los Angeles County community. At first glance, the bat appeared to be a common local free-tailed bat (*Tadarida brasiliensis*), but closer inspection indicated differences, although the bat belonged to a family with similar representatives in warm areas worldwide. After extensive study, I determined the specimen to be a Wrinkle-lipped bat *(Chaerephon pumila),* known throughout sub-Saharan Africa, Madagascar, and southern Arabia. Further research disclosed the transported bat’s African origin. This species supports experimental replication of Ebola virus without showing disease signs (*26*); the remainder of the carcass was immediately sent to a federal laboratory for Ebola virus tests, which proved negative. Several other viruses have also been isolated from the salivary glands of this species in Africa (*3*).

In June 1997, a woman was bitten by a bat hiding in clothing she was packing before an airline flight from Costa Rica to California. The live bat was restrained in a plastic bag during the flight; it was dead on arrival. The bat was negative for RABV and was identified as a Sinaloan Mastiff Bat (*Molossus sinaloae*), an insectivorous species in which RABV has been reported (*5*).

### Translocation for Confinement

Bats have been transported varying distances, sometimes worldwide, to be maintained in captivity as research animals, as live specimens in zoos or other exhibits, and as pets. Transport for research purposes is not noteworthy except in unusual circumstances. A Big Brown Bat in the incubational stages of rabies was among live bats sent from Canada to a laboratory in Germany, where the bat developed clinical rabies (*27*). Similarly, six Big Brown Bats that were incubating RABV were in a group sent from the United States to a laboratory in Denmark (*28*). However, recipient laboratories understood the risks and had taken necessary precautions.

RABV-infected individual bats of the tropical American common Vampire Bat *(Desmodus rotundus)* family have been reported throughout their geographic range, which extends from northern Mexico south to Chile and Uruguay (*8*,*29*). RABV has also developed in Vampire Bats after being transported to laboratories. In addition, during the 1970s, a group of these bats sent from Mexico to a laboratory in the United States presumably escaped en route, because only the empty shipping container arrived.

Increasing interest in bats has resulted in displaying of more varieties of these mammals, including Vampire Bats, to the public (*5*). One such display presented a problem I investigated in 1988 after four of eight Vampire Bats escaped their flight cage within a cavelike structure at a southern California zoo 1 month after their arrival from Mexico through a Texas supplier. Two escaped bats were found dead, possibly due to starvation or unusually cold weather. One dead bat had nearly escaped the building, and the other was outside. Neither bat was infected with RABV. The apparent escape route to the outside was through a fragile false cave ceiling, which could not be inspected. This ceiling may have contained the carcasses of the remaining two missing bats, possibly a male and a female. I found no bat bites on zoo animals and no bats or bat feces in likely hideaways in the zoo.

The large fruit-eating bats (genus *Pteropus)* live on land masses, including islands, from Madagascar, India, Southeast Asia, the East Indies, the Philippines, and Australia to the Samoan and Cook Islands of the South Pacific Ocean. They have been popular zoo attractions for many years. RABV was reported in a *Pteropus* in India (*8*), and RABV-related lyssaviruses were reported in four species of *Pteropus* and an insectivorous species in Australia, where two persons died of these infections (*30*).

Three additional viruses (*Paramyxoviridae* family) ascribed to *Pteropus* origin have proven pathogenic or fatal to people and domestic animals. Four species of Australian *Pteropus* bats in Queensland carry Hendra virus without developing symptoms. These bats disseminate virus in urine or placental fluid during birthing, and the virus is later ingested by pregnant horses that amplify the virus, which then spreads to people and causes a fatal pneumonia (13/20 horses were infected in a 1994 outbreak, which resulted in two human deaths) (*30*). The second virus, Menangle virus, is considered to be spread to pigs in Australia by the same four species of *Pteropus* bats, producing stillbirths with deformities in 1998 in 27% of litters, as well as an influenzalike illness in humans (*30*). The third virus, Nipah virus, identified in urine and saliva of *Pteropus* bats in Malaysia, apparently spreads the virus to pigs and destroyed that country’s swine industry in 1998. The virus spread from pigs to hundreds of industry workers; approximately 40% of these workers died of severe viral encephalitis caused by the agent (*31*).

Importation of fruit-eating bats has long been severely restricted to protect the fruit industry in the United States. The Egyptian Rousette bat (*Rousettus egyptiacus)* is a widespread Old World fruit bat that readily reproduces in captivity; thus colonies occur in some zoos. This species has been implicated in several viral infections in Africa (*3*). An error occurred in 1994, when thousands of these and other bat species were permitted entry into the United States for sale as pets or for exhibition (*28*); this procedural mistake resulted in a policy change to prevent recurrence. Antibodies to *West Nile virus* (WNV) had been reported in the *Rousettus* species in Uganda and Israel (*3*), and the virus had been isolated in India from the nearly indistinguishable *R. leschenaulti*, which overlaps geographically with *R.* e*gyptiacus* in Pakistan (*32*). The entry of *R.* *egyptiacus* into the United States in 1994 suggests a remote connection with the subsequent outbreak of WNV there, first observed 5 years later among captive and wild birds at a zoo in New York (*33*).

In 1997, two *R. egyptiacus* bats died with rabies-like symptoms in a Denmark zoo; they were later found to be infected with EBLV-1 subtype A, a RABV-related agent known to have caused deaths in European insectivorous bats and in humans. The two infected bats had arrived recently from a Netherlands zoo, where the source captive bat population subsequently was destroyed (*34*). A replacement colony was similarly destroyed after a bat originating from a Belgian zoo was also determined to be infected (*35*).

Persons concerned about sick and injured wildlife often try to rehabilitate disabled bats, sometimes transporting the animals a considerable distance from sites of discovery. Unfortunately, an average of 10% of disabled bats tested in North America are found to be infected with RABV, exposing those trying to rehabilitate the bats to rabies. If they have received preexposure rabies prophylaxis in advance, these persons are advised to take booster shots of vaccine; otherwise, they are advised to take both antirabies globulin as well as the full vaccine treatment.

Often, attempting to reverse the negative image of bats usually held by the public, persons trying to rehabilitate sick bats may suppress warnings of rabies hazards, doing both bats and the public a disservice. Moreover, to avoid the embarrassment of repeated exposures to rabid bats, some persons working in bat rehabilitation are known to arrange submission of rabies-suspect bats to a variety of different laboratories in different geographic areas, thus disguising the true history of the bat; this practice may protect the rehabilitator but prevent other persons or pets exposed earlier from receiving adequate antirabies management.

### Translocation for Release

Bats have been translocated and released in attempts to establish bat populations in new areas for reasons such as insect control and experimental study. Such efforts are sometimes supplemented by providing living quarters or shelters for bats ranging from elevated boxlike structures to tunnels. Before the knowledge that some insectivorous bats might be infected with rabies or other pathogens, bats were sometimes transported great distances over land or overseas and released in efforts to establish populations at the new location. Tomich (*13*) assembled historical records about the importation and release in rabies-free Hawaii of Asiatic Pipistrelle bats from Japan and free-tailed bats (*Tadarida brasiliensis)* from California during the late 1800s to establish bat populations for insect control purposes, but the attempts were evidently unsuccessful.

Observing that destruction of old-growth forests eliminated the tree hollow homes of Polish bats, Krzanowski (*36*) recommended the introduction into Poland of Red Bats and Hoary Bats from the United States because these species take shelter in tree foliage rather than hollows, and they migrate at the approach of cold weather rather than hibernate in tree cavities. However, rabies was discovered simultaneously in North American insectivorous bats, including these two species, discouraging further consideration of the proposal.

The homing abilities of bats have routinely been studied by transporting and releasing marked bats up to 805 km from their home roost, which is then monitored for the return of the marked bats (*37*). RABV infection has now been identified in 11 of the 12 North American species studied, and histoplasmosis is known in 6; RABV-related lyssavirus infections have been reported in 5 of 12 European species studied (*8*).

During World War II, field trials were conducted in the southwestern United States to determine the effectiveness of disseminating thousands of free-tailed bats *(T. brasiliensis)* in the air*,* each transporting a small time-activated fire bomb. The objective was to start thousands of simultaneous fires in adversary target areas, achieved after each bat had sought out a hideaway in various available structures (*38*). As a participant in the project, I observed that each bomb or dummy bomb, attached by a short string and surgical clip to the bat’s abdominal skin, was disengaged after the bat alighted in a refuge and chewed through the string. Thousands of bats were transported <1,609 km distant from source bat caves in Texas and New Mexico to test areas in California, New Mexico, and Utah. Frequently, the tests were postponed, and the freshly captured bats were released unencumbered at or near test sites. Unknown at the time, RABV is now known to occur in 0.5% of bats in the source caves (*8*), so the virus was almost certainly translocated with the bats. *H. capsulatum,* the causative fungus of histoplasmosis, also has been isolated from these bats and their guano in the source caves, but neither bats nor guano have yielded the agent in extensive surveys in California, which is regarded as free of the fungus; no cases of indigenous origin have been detected (*8*).

## Discussion

Bats and the pathogenic organisms they sometimes harbor are being transported by humans within and between continents, and sometimes these transported bats escape. Because bats reproduce slowly (usually only one or two offspring are produced annually by a female), the chances of successful introduction of the species are minimized. Populations would more likely develop should large numbers be freed in places favorable to survival. Although a single escaped bat might not survive long or reproduce, it would seek shelter in places frequented by local bats to which it might transmit pathogens. As has been observed, introduced pathogens include RABV, other lyssaviruses, or various other agents.

Vampire Bats can be especially problematic in view of their possible colonization in warm climates and their dependence on a diet of blood, thus necessitating their biting vertebrates, including man and domestic animals. As reported, in addition to their known role as biologic vectors of rabies to humans and domestic animals and surra *(Trypanosoma evansi)* to horses and cattle, Vampire Bats can also be temporary biologic as well as mechanical vectors of *Venezuelan equine encephalomyelitis virus* and foot-and-mouth disease. They are likely effective mechanical vectors if not biologic vectors of any bloodborne pathogen, including the AIDS virus (*29*). Various species of fruit-eating bats are infected at times with pathogens destructive to other bats, humans, and domestic animals. However, their entry to many areas is restricted due to concern that their escape would lead to populations destructive to fruit crops.

Accidental or planned translocations of bats between land masses happens almost certainly with far greater frequency than is reported. Such events can be embarrassing, and although incidents that result in successful containment are more likely to be reported, failed efforts can remain unpublicized. Relevant reporting requirements do not exist. Personnel involved in the various described incidents generally have performed very well in efforts to resolve the problems, often with immediately contrived solutions. Inspectors at entry centers are usually exceptionally competent because they must cover a broad array of subject areas, but their competency must be taxed at times. For example, most bats are exceptionally adept at avoiding capture, and even bat scientists with special equipment frequently are outmaneuvered. Some inspectors contact specialists for help in emergencies, but help is not always available or is displaced by previous commitments and economic necessities. Previous contractual arrangements with institutions such as universities, natural history museums, zoos, or specialized commercial services could dispel most relevant problems, including funding, and maintain program continuity. Unaffiliated specialized personnel would be expected to maintain or acquire relevant competency, but incidents, such as those cited here, show some lapses. Ideally, the services of a bat expert are required. For example, if bats are to be excluded from any vehicle of conveyance, the usual procedures and equipment should be reviewed by responsible persons very familiar with bats, their capabilities, their capture, their confinement, and their exclusion in order to recognize flaws that permit bats to be transported. Thus, experts can help establish and maintain more effective programs.
